# A rare *Wohlfahrtiimonas* strain linked to human infection

**DOI:** 10.3389/fcimb.2025.1609676

**Published:** 2025-06-23

**Authors:** Peiying Huang, Chao Yang, Meifang Lin, Guanghui Song, Ping-Hua Qu, Li Chen

**Affiliations:** ^1^ Emergency Department, Guangdong Provincial Hospital of Chinese Medicine, The Second Affiliated Hospital of Guangzhou University of Chinese Medicine, Guangzhou, China; ^2^ The Second Clinic Medical College, Guangzhou University of Chinese Medicine, Guangzhou, China; ^3^ Department of Laboratory, The Second Affiliated Hospital of Guangzhou Medical University, Guangzhou, China; ^4^ Ultrasound Department, Guangdong Provincial Hospital of Chinese Medicine, The Second Affiliated Hospital of Guangzhou University of Chinese Medicine, Guangzhou, China; ^5^ School of Medicine, Foshan University, Foshan, China

**Keywords:** Wohlfahrtiimonas, bacterium, sepsis, 16S rRNA gene, whole-genome sequencing

## Abstract

*Wohlfahrtiimonas* is an infrequently encountered Gram-negative bacterium capable of infecting humans and, in severe instances, precipitating sepsis. Presently, three species within the *Wohlfahrtiimonas* genus have been identified, with *Wohlfahrtiimonas chitiniclastica* being the sole species implicated in human infections. To date, there has been only one documented case of human infection with *W. chitiniclastica* in China. In this study, we present an additional case of human infection with a *Wohlfahrtiimonas* species. Notably, through 16S rRNA gene sequencing and whole-genome sequencing, the strain was identified as an unclassified species closely related to *W. chitiniclastica* DSM 18708^T^.

## Introduction

*Wohlfahrtiimonas* is a zoonotic, Gram-negative bacterium first isolated from the larvae of the parasitic fly *Wohlfahrtia magnifica*, a species known for causing myiasis through larval deposition in open wounds (Tóth et al., 2008). This bacterium is associated with human infections, including bacteremia and sepsis, with reported cases spanning diverse regions: 21 cases in Europe, 15 in the United States, 6 in Asia, and individual cases in Africa and Australia (Kopf et al., 2024; Yuan et al., 2025). The identified species of *Wohlfahrtiimonas* comprise *Wohlfahrtiimonas chitiniclastica*, *Wohlfahrtiimonas larvae*, and *Wohlfahrtiimonas populi* (Lee et al., 2014; Li et al., 2017; Yuan et al., 2025). To date, *W. chitiniclastica* remains the sole species of *Wohlfahrtiimonas* isolated from human hosts (Yuan et al., 2025).

Currently, documented instances of *Wohlfahrtiimonas* infections in humans within China are infrequent. In this study, we document the isolation of *Wohlfahrtiimonas* from the wound of a patient with sepsis in China. The identification based on whole-genome sequencing indicates that this strain is represented as an unclassiffed species which is phylogenetically closely related to *W. chitiniclastica* DSM 18708^T^. Moreover, in contrast to conventional beliefs of *Wohlfahrtiimonas*, our experimental findings verified that it is a facultative anaerobic bacterium.

## Clinical case

**Figure 1:**
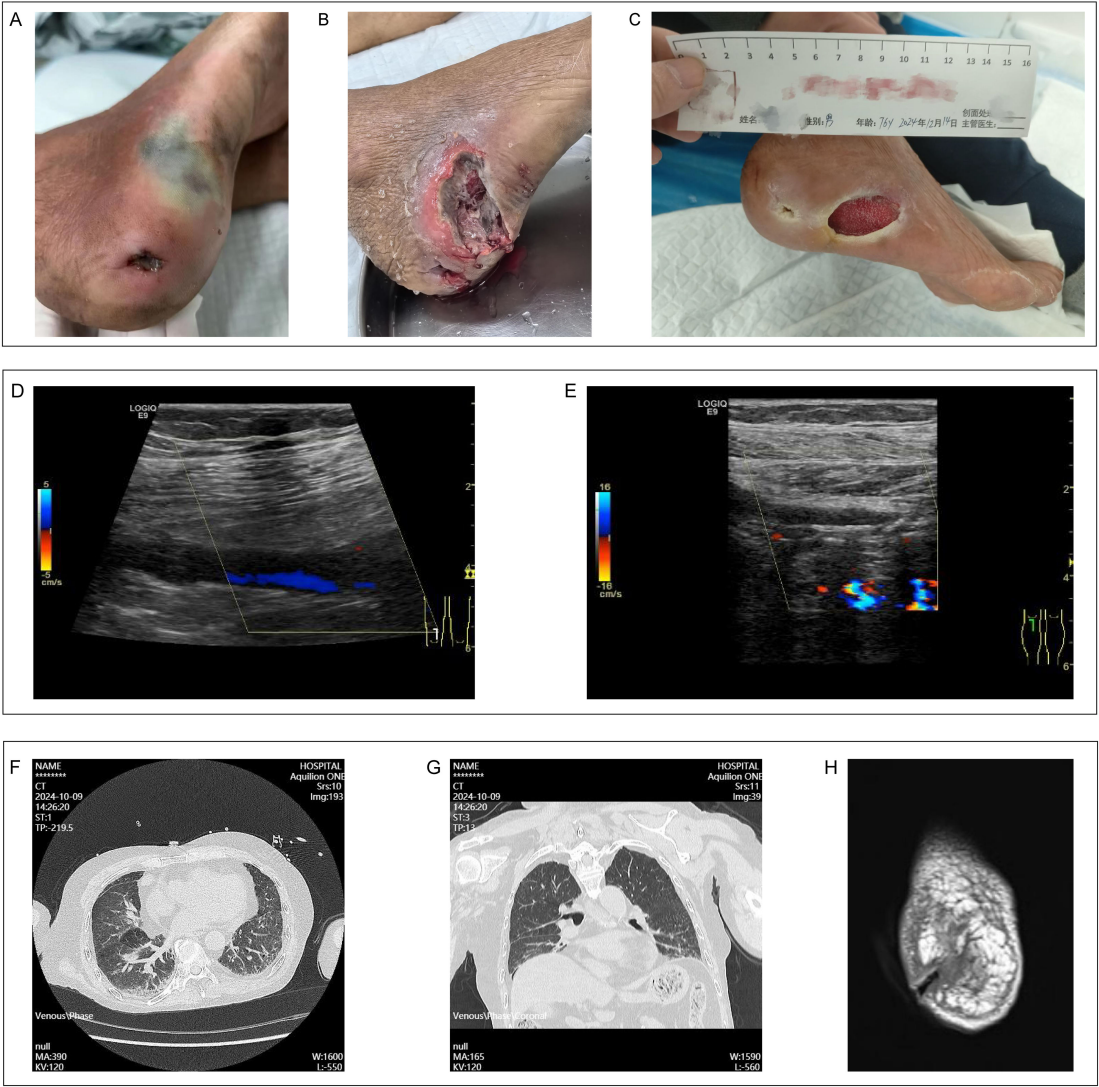
Clinical data for patient. **(A)** The condition of the wound on the patient's right foot admission. **(B)** The status of the right foot wound following incision and disinfection. **(C)** The healing process of the patient's right foot wound. **(D)** Color Doppler ultrasound imaging of thrombosis in the popliteal vein of the right lower limb. **(E)** Color Doppler ultrasound imaging indicating stenosis in the popliteal artery of the right lower limb. **(F, G)** Chest CT findings obtained subsequent to the patient's admission. **(H)** MRI findings pertaining to the wound on the right foot upon the patient's admission.

A local 76-year-old male farmer presented with an 8-year history of recurrent bilateral lower extremity edema and pain, accompanied by impaired dorsiflexion of the right foot and a 7-day history of fever in Guangdong Provincial Hospital of Traditional Chinese Medicine, Guangzhou (the capital city of Guangdong province, China). Upon admission, the patient exhibited a fever of 38.8°C, lethargy, productive cough, bilateral lower extremity pain and swelling, intermittent claudication, and difficulty with dorsiflexion of the right foot. Clinical examination revealed a 1 cm × 1.5 cm ulceration and a 3 cm × 3.5 cm ecchymosis on the lateral aspect of his right foot (**Figure 1A**).

The patient exhibited multiple comorbidities, including vasculitis, deep vein thrombosis of the right external iliac vein (inguinal segment), common femoral vein, superficial femoral vein, popliteal vein (**Figure 1D**), peroneal vein, and soleal muscle vein, and lower extremity arterial occlusion in the right popliteal artery (**Figure 1E**), anterior tibial artery (middle and distal segments), and dorsalis pedis artery. Additionally, the patient was diagnosed with Stage 4 chronic kidney disease, steroid-induced diabetes, severe osteoporosis, chronic heart failure, and hypertension. Over the past 8 years, the patient lived in conditions of poor sanitation and hygiene and has intermittently received treatment with methylprednisolone, hydroxychloroquine, and cyclophosphamide.

Upon admission, a computed tomography scan of the chest identified bilateral interstitial pneumonia (**Figures 1F, G**). Additionally, magnetic resonance imaging of the foot revealed extensive soft tissue edema in the right foot, a disruption of the tissue at the heel, degenerative alterations in the bones of the right foot, and a minor effusion in the right ankle joint (**Figure 1H**). Laboratory analyses indicated an elevated hypersensitive C-reactive protein level of 177.11 mg/L, a white blood cell count of 29.53 × 10^9^/L, a neutrophil count of 26.19 × 10^9^/L, fibrinogen concentration of 8.71 g/L, and a procalcitonin level of 1.65 ng/mL. Considering the patient’s overall condition, a Sequential Organ Failure Assessment (SOFA) score of 6 was assigned which meet the sepsis diagnosis.

Empirical intravenous therapy with piperacillin-tazobactam was commenced to address the infection. Sputum, wound swab, and venous blood samples were collected for microbial culture and identification prior to the administration of antibiotics. On the second day of hospitalization, the patient exhibited dyspnea with oxygen saturation levels fluctuating between 80% and 90%. Thus, orotracheal intubation was performed at the bedside, followed by the initiation of mechanical ventilation. Subsequently, bronchoalveolar lavage fluid was obtained for further pathogen detection.

Lab tests identified *Wohlfahrtiimonas*, *Staphylococcus haemolyticus*, and *Bacteroides fragilis* in the wound swab from the patient’s right heel. Blood cultures revealed the presence of *Staphylococcus epidermidis*. The bacterial culture of bronchoalveolar lavage fluid (BALF) indicated normal upper respiratory microbiota with a minor presence of yeast-like fungi, while next-generation sequencing (NGS) of the BALF further identified *Klebsiella pneumoniae* (752 sequences), *Acinetobacter baumannii* (59 sequences), and *Streptococcus anginosus group* (5396 sequences).

During subsequent management, an incision and drainage procedure were conducted on the patient’s right dorsal foot wound (**Figure 1B**), and the antibiotic regimen was enhanced with the addition of voriconazole and linezolid to control the infection. The patient was extubated on the seventh day of hospitalization, transferred from the intensive care unit on the ninth day, and discharged on the fourteenth day, with the infection successfully managed. One month after discharge, the patient’s right foot wound showed signs of healing and was being prepared for skin grafting (**Figure 1C**).

It is important to highlight that the identification of *Wohlfahrtiimonas* and its associated infections in humans are exceedingly rare occurrences in mainland China. Consequently, we undertook a series of experiments on this particular strain to enhance understanding of its characteristics.

## Method and materials

The wound swab from the patient’s foot wound was inoculated onto Columbia blood agar, Chocolate agar and MacConkey agar at 37°C incubator with 5% CO_2_ and 18%-21% O_2_ environment. One strain, designated Woh60147, was successfully identified by matrix-assisted laser desorption/ionization-time-of-flight mass spectrometry (MALDI-TOF MS, Bruker, Bremen, Germany), but not with the VITEK 2 Compact system (BioMérieux, Lyon, France). This strain was isolated following a 24-hour incubation period. Then, we performed a Gram staining on this strain. The protein molecular mass spectrogram of the strain was acquired by MALDI-TOF MS with the MBT Compass Library Revision L (2020) and covering 3239 species/entries (9607 MSP) and subsequently compared with the spectra of known bacterial species in the database provided by the manufacturer.

To further evaluate the strain’s resistance to commonly utilized clinical antibiotics, a drug susceptibility test was conducted employing the VITEK 2 Compact system. Antimicrobial susceptibility breakpoints were interpreted in accordance with the Clinical and Laboratory Standards Institute (CLSI) M100 guidelines for non-Enterobacterales species (Institute C a L S, 2024). Additionally, to examine the strain’s growth under different oxygen concentrations, it was cultured under anoxic (0% O_2_) and microaerophilic (6% O_2_) conditions for a duration of 48 hours, during which its growth trajectory was monitored.

In order to investigate the phylogenetic features of the isolated strain Woh60147, 16S rRNA gene sequencing, whole-genome sequencing, and neighbor-joining phylogenetic tree was performed.

The upstream primer utilized for 16S rRNA gene sequencing is designated as 27F (5’-AGAGTTTGATCMTGGCTCAG-3’), while the downstream primer is identified as 1492R (5’-GGTTACCTTGTTACGACTT-3’). Detailed parameters for the specific PCR amplification are presented in **Table 1**.

**Table 1:** PCR amplification parameters for 16S rRNA gene sequencing.

Program	Temperature	Duration
Pre‐denaturation	95°C	5 min
32 cycles		
Denaturation	94°C	30s
Annealing	52°C	30s
Extension	72°C	1min and 30s
Cycle ended		
Re-extension	72°C	7min

Genomic DNA from the strain Woh60147 was extracted employing the Steadyu Bacterial Genomic DNA Extraction Kit. The whole genome sequencing was conducted using the Illumina Novaseq 6000 high-throughput sequencer. The sequencing process involved several key steps: initial filtering of raw data was performed using fastp v0.20.1 software; assembly was carried out with SPAdes v3.15.3; contigs shorter than 500 bp were excluded using seqkit2 software; and the genome’s completeness, contamination rate, and N50 were assessed using CheckM2 v1.0.1. Genomes with completeness below 90% or contamination rates exceeding 5% were excluded from the study. Following data filtration, species classification was executed using GTDB-Tk v2.1.1, and genome annotation for qualified samples was performed using Prokka v1.13.3 software.

Subsequently, we employed the Virulence Factors of Pathogenic Bacteria (VFDB) database (http://www.mgc.ac.cn/VFs/), applying similarity and coverage thresholds of 60%, to identify the virulence genes of the strain. On the other hand, in the initial phase of the experiment, although, drug susceptibility tests were performed, to achieve a more thorough understanding of the drug resistance genes present in this strain, we employed the ResFinder database (https://cge.cbs.dtu.dk/services/ResFinder/) and the Comprehensive Antibiotic Resistance Database (CARD) (https://card.mcmaster.ca/), with the same thresholds, to screen for the strain’s drug resistance genes.

The construction of the phylogenetic tree was accomplished utilizing the GTDB-Tk v2 and IQ-TREE v2 software. Initially, all genomic sequence files of the strain Woh60147, formatted in fna, were uploaded to the online server. Within the GTDB-Tk v2, which operates in a Python environment, the ‘identify’ function within the ‘classify wf’ workflow was employed to detect and screen single-copy marker genes in the genome. Subsequently, the ‘align’ function facilitated multiple sequence alignment. The resulting output file was then decompressed into a protein sequence file using the ‘gunzip’ command. Following this, the IQ-TREE v2 was employed, where the command ‘iqtree-s gtdbtk.bac120.user_msa.fasta-m MFP’ was executed to determine the optimal tree-building model. Upon execution, the best-fit model result was copied, and the command ‘iqtree-s gtdbtk.bac120.user_msa.fasta-m best-fit_model-bb 1000-bnni-redo’ was executed to initiate the construction of the phylogenetic tree.

## Results

**Figure 2:**
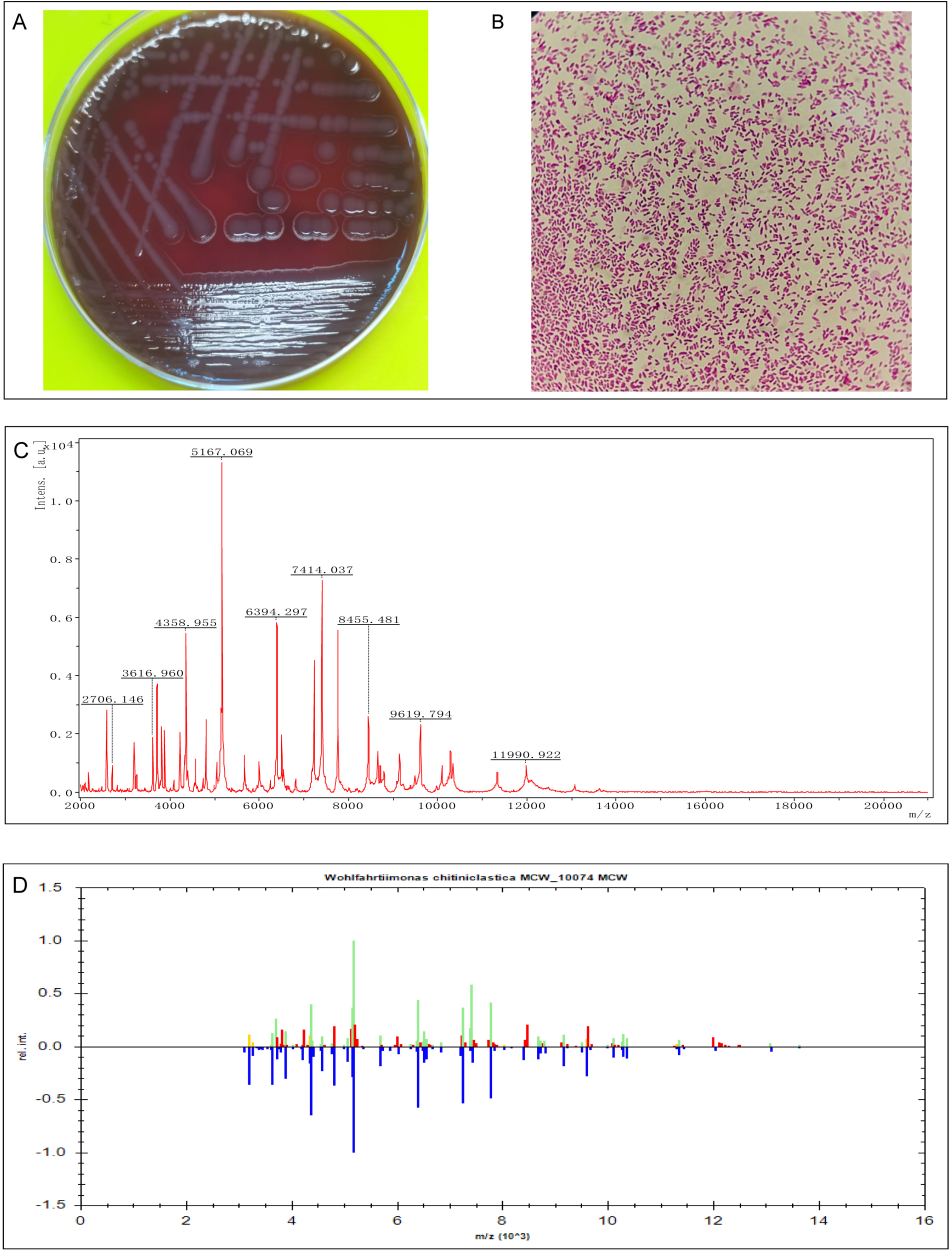
Isolation and identification of *Wohlfahrtiimonas* strain Woh60147. **(A)** Bacterial colonies on Columbia blood agar after cultured at 37°C in the presence of 5% CO_2_ and 18-21% O_2_ for 24h. **(B)** The result of Gram staining of this strain Woh60147 under the microscope. **(C)** The spectrogram with protein molecular mass of the strain acquired by MALDI-TOF MS. **(D)** Spectra comparison of the strain Woh60147 with that of known strains in a database matched to *Wohlfahrtiimonas chitiniclastica* MCV_10074 MCV with a high confidence level.

In the conventional incubator (18%-21% O_2_), the strain grew well in Columbia blood agar, Chocolate agar and MacConkey agar. The colony was gray, opaque, round, smooth, with a well-defined edge of more than 2mm diameter (**Figure 2A**). Microscopic examination following Gram staining showed that strain Woh60147 was Gram-negative short rod-shaped bacteria (**Figure 2B**). The protein molecular mass spectrogram of the strain was presented in **Figure 2C**. According to the spectra comparison, the strain Woh60147 was matched to *W. chitiniclastica* MCV_10074 (**Figure 2D**), achieving a score of 2.379.

**Figure 3:**
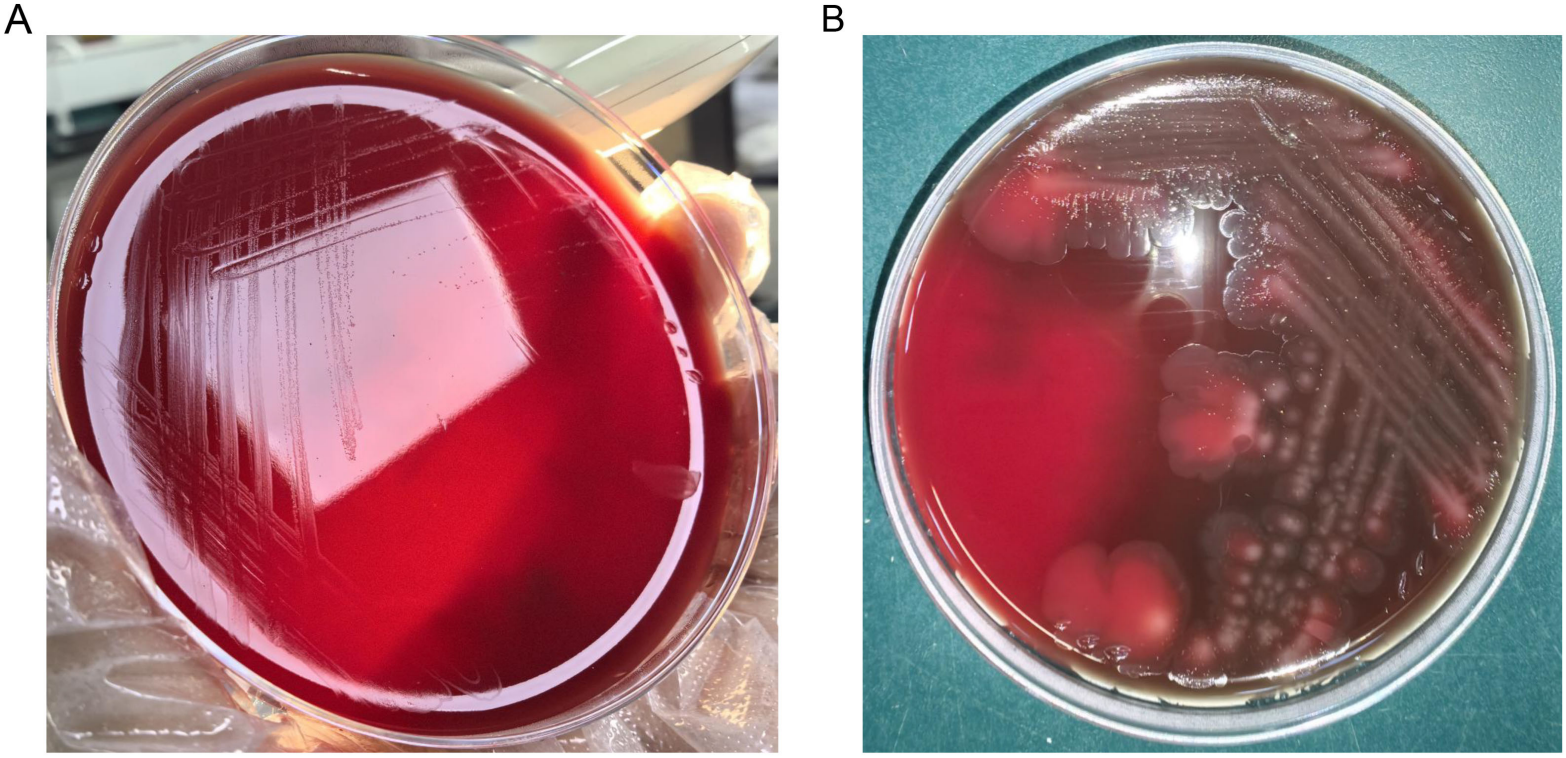
**(A)** Bacterial colonies on Columbia blood agar after being cultured at 37°C in the presence of 5% CO_2_ and anaerobic environment for 48h. **(B)** Bacterial colonies on Columbia blood agar after being cultured at 37°C in the prlesence of the 5% CO_2_ and 6% O_2_ for 48h.

The antimicrobial susceptibility results based on CLSI M100 also showed that the strain was sensitive to 14 antibiotics commonly used in clinical settings. The result of antimicrobial susceptibility testing for Woh60147 was shown in **Table 2**. Interestingly, the strain demonstrated the ability to grow in an anaerobic environment, forming pin-point colonies after 48 hours of incubation; while in a microaerophilic environment (6% O_2_), its growth pattern was comparable to that observed in conventional culture (18%-21% O_2_) (**Figure 3**).

**Table 2:** Antimicrobial susceptibility test results for strain Woh60147.

Antibiotic	MIC	Sensitivity
Ciprofloxacin	≤0.25 ug/mL	Sensitive
Meropenem	≤0.25 ug/mL	Sensitive
Imipenem	≤0.25 ug/mL	Sensitive
Tobramycin	≤1.00 ug/mL	Sensitive
Ticarcillin/clavulanic acid	≤8.00 ug/mL	Sensitive
Ceftazidime	≤0.12 ug/mL	Sensitive
Cefoperazone/sulbactam	≤8.00 ug/mL	Sensitive
Minocycline	≤1.00 ug/mL	Sensitive
Levofloxacin	≤0.12 ug/mL	Sensitive
Doxycycline	≤0.50 ug/mL	Sensitive
Tigecycline	≤0.50 ug/mL	Sensitive
Cotrimoxazole	≤1.00 ug/mL	Sensitive
Amikacin	=4.00 ug/mL	Sensitive
Colistin	≤0.50 ug/mL	Sensitive

MIC, Minimum Inhibitory Concentration.

According to the analysis, the genome size of strain Woh60147 is 1,789,670 base pairs, comprising 275 contigs and exhibiting a GC content of 43.7%. The genome encodes 1,659 coding sequences, 3 ribosomal RNA genes, 3 repeat regions, 41 transfer RNA genes, and 1 transfer-messenger RNA gene. Notably, the strain Woh60147 harbors 4 virulence-associated genes: gtrB, htpB, katA, and pilT. However, screening with ResFinder and CARD did not identify any antibiotic resistance genes within the genome of the strain Woh60147.

**Figure 4:**
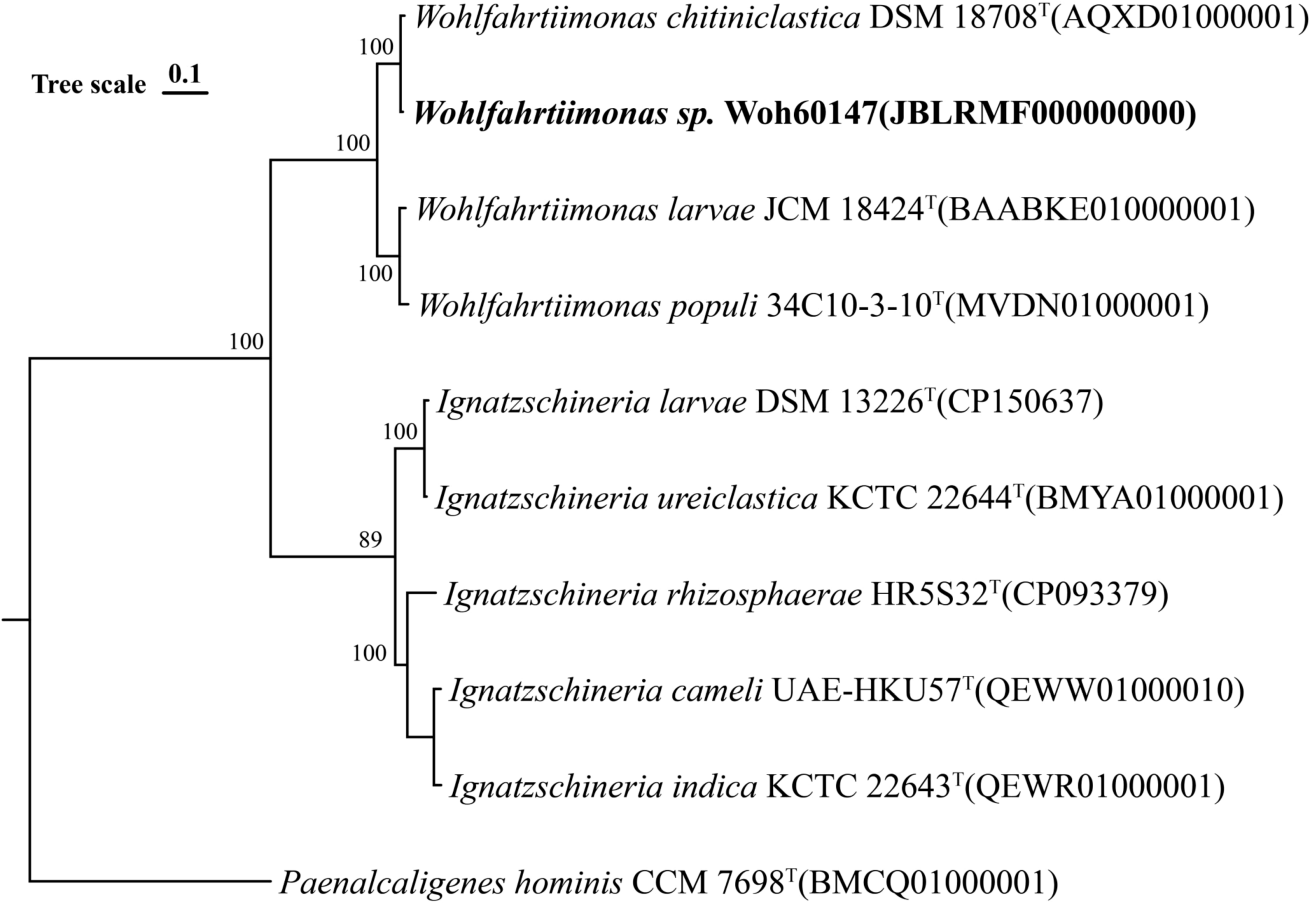
Neighbor-joining phylogenetic tree based on whole-genome sequencing of the strain (Woh60147) isolated from the right foot wound from the septic patient.

Results of the 16S rRNA gene sequence of 1413 nucleotide showed 100% identity to *W*. *chitiniclastica* DSM 18708 ^T^. Neighbor-joining phylogenetic tree based on whole-genome sequencing also showed the strain Woh60147 was closely related to *W*. *chitiniclastica* DSM 18708 ^T^ (**Figure 4**). However, the average nucleotide identity (ANI) between the genomes of the two strains was 93.06%, and the average digital DNA-DNA hybridization value between them was 46.4%, both of which were lower than the threshold values (95%-96% average nucleotide identity and 70% digital DNA-DNA hybridization) used for delineating prokaryotic species (Chun et al., 2018). Therefore, this finding suggests that strain Woh60147 represents an unclassiffed species that is phylogenetically closely related to *W. chitiniclastica*. The 16S rRNA sequencing date (accession no. PV123151) and the whole-genome sequencing data (accession no. JBLRMF000000000) were deposited into the National Center for Biotechnology Information (NCBI) under the GenBank accessions.

## Discussion

Infections caused by *Wohlfahrtiimonas* predominantly manifest in individuals with compromised health status, such as those suffering from chronic skin ulcers, diabetes mellitus, cardiovascular disease, a history of alcohol abuse, immunosuppression, or inadequate sanitary conditions (Kopf et al., 2024). This susceptibility may be attributed to the host’s relatively diminished immune function and the elevated risk of encountering pathogenic bacteria in the environment. Or in other words, *Wohlfahrtiimonas* may act as an opportunistic pathogen, as previously documented (Katanami et al., 2018; Bueide et al., 2021; Kopf et al., 2021). In the present case, the patient exemplifies this susceptible demographic.

In contrast to previously documented cases, this report presents a human infection attributed to a novel strain of *W. chitiniclastica*. Although 27 sequences of *W. chitiniclastica* have been identified to date (Wohlfahrtimonas Strains Genome), the identification of new strains, along with the investigation of their intrinsic antibiotic susceptibility and the host’s infection response, are critical components in the advancement of clinical infectious disease research.

Current case on the antimicrobial resistance profile of *Wohlfahrtiimonas* suggest that this bacteria exhibits susceptibility to a range of antibiotics, including β-lactams, sulfamethoxazole-trimethoprim, tetracycline, and fluoroquinolones, while demonstrating resistance to aminoglycosides (such as amikacin and tobramycin) and fosfomycin (Chavez et al., 2017; Snyder et al., 2020; Bueide et al., 2021; Harfouch et al., 2021; Kopf et al., 2021, 2024). In this case, no resistance to the antibiotics tested was observed for the isolated *W. chitiniclastica*. The patient initially received treatment with piperacillin-tazobactam. After that, voriconazole and linezolid were administered to mitigate other potential polymicrobial infections. This therapeutic approach successfully managed the patient’s infection.

An additional noteworthy characteristic of this strain is its ability to proliferate under microaerobic or even anaerobic conditions, contrary to earlier research indicating that *W. chitiniclastica* is a strict aerobe (Kopf et al., 2022). This observation aligns with the findings of Ahmad et al., Nogi et al., and Chevez et al., who suggested that *W. chitiniclastica* functions as an obligate anaerobe (Nogi et al., 2016; Chavez et al., 2017; Ahmad et al., 2022). Consequently, in certain clinical instances of deep tissue infections, the possibility of *W. chitiniclastica* infection should be considered.

Alongside *Wohlfahrtiimonas*, a variety of pathogenic microorganisms were identified in the patient’s blood, pulmonary secretions, and wound. The presence of these multiple pathogens complicates the determination of the definitive etiology of the patient’s sepsis. While *Wohlfahrtiimonas* is recognized as a pathogenic organism, determining its independent contribution to this case of sepsis remains challenging (Kopf et al., 2024). Nonetheless, prior case reports have unequivocally demonstrated instances of bacteremia and sepsis attributable solely to *Wohlfahrtiimonas*, suggesting that this bacterium can cause severe infections under certain conditions (Almuzara et al., 2011; Harfouch et al., 2021). Consequently, as a rare potential pathogen, *Wohlfahrtiimonas* necessitates vigilant clinical monitoring, especially in patients with open wounds, diabetes, or compromised immune systems.

As of now, reports of *Wohlfahrtiimonas* infections in China are scarce. Beyond the identification of *W. chitiniclastica* in the ulcerative wound on a male patient’s left foot, other instances have involved the isolation of *W. chitiniclastica* from Chrysomya megacephala and the pancreas of zebras (Cao et al., 2013; Zhou et al., 2016; Yuan et al., 2025). Nevertheless, the limited documentation of *Wohlfahrtiimonas* infections does not inherently indicate a low occurrence of these cases in the region. Rather, it is likely that the limited adoption of primary diagnostic techniques for *Wohlfahrtiimonas* infections, such as 16S rRNA gene sequencing and whole-genome sequencing, in numerous clinical laboratories across China may contribute to an underestimation of the pathogen’s prevalence within the population. In comparison to conventional pathogen identification techniques, NGS demonstrates a superior detection rate (Chen et al., 2020). Similar to the findings of the present study, the VITEK 2 Compact system was unable to identify the strain Woh60147. Nevertheless, the integration of NGS technology for pathogen detection into China’s medical insurance system remains pending, limiting its widespread adoption across the country (Zhang et al., 2025). In additionally, in many urban and rural areas of China, the identification of infectious microorganisms predominantly relies on the clinical assessment of patients’ symptoms and signs due to the lack of comprehensive microbiological testing (Currie et al., 2014). Therefore, it is imperative to reduce the costs associated with emerging identification technologies and to promote their extensive utilization. In particular, the incorporation of these technologies in cases of unexplained infections may enhance diagnostic accuracy for both known and unknown pathogens, thereby potentially improving clinical outcomes for certain patients.

## Data Availability

The datasets presented in this study can be found in online repositories. The names of the repository/repositories and accession number(s) can be found below: https://www.ncbi.nlm.nih.gov/, PV123151 https://www.ncbi.nlm.nih.gov/, JBLRMF000000000.
